# Py–FTIR–GC/MS Analysis of Volatile Products of Automobile Shredder Residue Pyrolysis

**DOI:** 10.3390/polym12112734

**Published:** 2020-11-18

**Authors:** Bin Yang, Ming Chen

**Affiliations:** School of Mechanical Engineering, Shanghai Jiao Tong University, No. 800, Dongchuan Rd., Shanghai 200240, China; robinshjt@sjtu.edu.cn

**Keywords:** automobile shredder residue (ASR), pyrolysis, thermogravimetric analysis, gas chromatograph, gas chromatography mass spectrometry, Fourier transform infrared spectrometry

## Abstract

Automobile shredder residue (ASR) pyrolysis produces solid, liquid, and gaseous products, particularly pyrolysis oil and gas, which could be used as renewable alternative energy resources. Due to the primary pyrolysis reaction not being complete, the yield of gaseous product is low. The pyrolysis tar comprises chemically unstable volatiles before condensing into liquid. Understanding the characteristics of volatile products will aid the design and improvement of subsequent processes. In order to accurately analyze the chemical characteristics and yields of volatile products of ASR primary pyrolysis, TG–FTIR–GC/MS analysis technology was used. According to the analysis results of the Gram–Schmidt profiles, the 3D stack plots, and GC/MS chromatograms of MixASR, ASR, and its main components, the major pyrolytic products of ASR included alkanes, olefins, and alcohols, and both had dense and indistinguishable weak peaks in the wavenumber range of 1900–1400 cm^−1^. Many of these products have unstable or weaker chemical bonds, such as =CH–, =CH2, –C=C–, and –C=CH2. Hence, more syngas with higher heating values can be obtained with further catalytic pyrolysis gasification, steam gasification, or higher temperature pyrolysis.

## 1. Introduction

The dismantling and recycling of end-of-life vehicles (ELVs) is not only an important way to save resources and realize sustainable development but also bears huge social and environmental responsibility. As of the end of 2019, China’s car ownership exceeded 260 million. According to the international scrap ratio of 4–6%, the quantity of ELVs in China will be large in the future. Automobile shredder residue (ASR) accounts for about 20–25% of a vehicle’s weight [1,2,3,4,5,6] and consists of plastic, textile, leather, rubber, foam, wood, paper, glass, sand, metals, and other materials [3,4,5,6,7,8]. Organic components account for about 60–85% of ASR [9,10], for which thermal conversion technology represents a viable resource recovery process. Pyrolysis and gasification have gradually become the main means to dispose of ASR, since they can reduce the volume and quality of landfill with lower cost, while energy recovery can be carried out simultaneously [11,12,13]. ASR pyrolysis produces solid, liquid, and gaseous products, particularly pyrolysis oil and gas, which could be used as renewable alternative energy resources.

The yields and characteristics of ASR pyrolytic products depend on the feed (e.g., fractions of organic versus inorganic), temperature, residence time, and carrier gases used [12]. There is a great range of variations possible, and the values used in a given process design must be chosen depending on which products are the process targets. Harder and Forton analyzed the variations in the physical characteristics of ASR, provided a review of the current technical developments in pyrolysis processes, and emphasized that the energy content of ASR is crucial to the design of a thermal process [14]. Zolezzi et al. evaluated the performance and product yields of conversional and fast pyrolysis of ASR at different temperatures [12]. It proved that the gas yield in conventional pyrolysis was higher than in fast pyrolysis, and higher heating values (HHVs) of gas increased with increasing temperature. HHV gas is also the optimal pyrolytic product of our research project funded by the National Natural Science Foundation of China No. 51675343. Extensive experimental research into ASR pyrolysis by other authors [15,16,17,18,19,20] has shown CO, CO_2_, H_2_, and light hydrocarbons (C1–C4, such as CH_4_, C_2_H_2_, C_2_H_4_, C_2_H_6_, C_3_H_6_) as the dominant constituents of HHV gas. We also studied the pyrolytic product yields and characterizations of gaseous products (H_2_, CO, CO_2_, CH_4_, C_2_H_4_, C_2_H_6_, C_3_H_6_ and C_3_H_8_), which were analyzed in laboratory-scale non-isothermal pyrolysis experiments at finished temperatures of 500, 600, and 700 °C [21]. Moreover, we found that the pyrolytic reaction was insufficient in primary ASR pyrolysis, and the yield and calorific value of HHV gas are limited. Since the yield of gaseous product is low, however, the yield of tar and char are always higher. Our previous results show that tar and gas accounted for 39.68 and 19.76% of the pyrolysis product when the pyrolysis temperature was set to 600 °C [21].

Catalytic pyrolysis gasification [22,23,24], steam gasification [25,26], and higher temperature pyrolysis [27] are effective methods of increasing HHV gas production. Aiming to improve the yields of syngas and hydrogen, a two-stage pyrolysis process with catalysts was designed in our research project. Before the polymer organic mixtures pyrolyzed in the first-stage reactor condensed into tar, secondary pyrolysis was expected to continue under the action of catalysts. Lin et al. [28] presented an analysis of the catalytic gasification of ASR for the generation of high-purity hydrogen in a lab-scale fixed-bed downdraft gasifier using 15 wt.% NiO/Al_2_O_3_ catalysts at 760–900 K. They verified that the NiO/Al_2_O_3_ catalysts can increase the output of hydrogen and CO, but did not explain how the catalyst affects the secondary reaction of pyrolysis intermediate products. The main challenges of implementing and improving catalytic gasification technology were to select or develop suitable catalysts for enhancing synthesis gas yield. Most pyrolysis tar is also volatile before condensing into liquid, and its chemical state is very unstable. Understanding the characteristics of volatile products will aid the design and improvement of subsequent processes. Thus, the purpose of this study was to analyze the chemical compositions and yields of volatile products of primary ASR pyrolysis.

Thermogravimetric analysis (TGA), coupled with Fourier transform infrared spectrometry (FTIR) and gas chromatography–mass spectrometry (GC/MS), is a good means by which to explore not only the weight loss characteristics and kinetics parameters of the thermal pyrolysis process but also identify the gaseous byproduct generated in real time. Kai et al. [29] investigated the effects of the interaction between rice straw (RS) and high-density polyethylene (HDPE) on the evolution of volatile species and their distributions during the co-pyrolysis of RS and HDPE by TG–FTIR–MS. Wu et al. [30] analyzed the pyrolysis and co-pyrolysis behaviors of polyethylene (PE), polystyrene (PS), and polyvinyl chloride (PVC) under N_2_ atmosphere by TG–FTIR. Zhang et al. [31] investigated the thermal decomposition of six representative components of municipal solid waste (MSW, including lignin, printing paper, cotton, rubber, PVC, and cabbage). Surjit Singh et al. [32] reported the characterization and assessment of the volatile species evolved during the thermal degradation of several waste materials, biomass wood waste, refuse derived fuel (RDF), waste plastic, and tire by use of TGA–FTIR and TGA–MS. Guo et al. [33] analyzed gas emissions from the thermo-oxidative process of plastic ASR under different N_2_/O_2_ volume ratios using TG–MS analysis, but only the production changes of small molecular gases under different reaction conditions were considered, such as H_2_, CH_4_, NH_3_, HCN, NO, NO_2_, CO_2_, and SO_2_. Mayyas et al. [34] studied the effect of the TiO_2_ catalyst on the pyrolyzed byproducts of industrial plastic waste (i.e., automotive shredder residue, ASR) with TG–FTIR–GC/MS. In our study, in the TGA experiments for ASR, each main component (plastic, textile, leather, rubber, foam) and artificial mixture was examined with a thermogravimetric analyzer under non-isothermal conditions. Simultaneously, the chemical compositions of volatile products of the primary ASR pyrolysis and its main components were detected and identified by TG–FTIR–GC/MS.

## 2. Experimental Procedure and Specimens

### 2.1. Experimental Specimens

The ASR specimens studied in this work were obtained from the Zhangjiagang Huaren Resources Recycling Co., Ltd. (Zhangjiagang, Jiangsu Province, China), a domestic automobile dismantling enterprise. Reusable components with market value and particularly valuable material fractions such as batteries, air bags, tires, and catalytic converters are usually removed from ELVs. The remaining parts and the hulks are reduced to small pieces, most of the metal fraction is sorted using magnetic separation and eddy current separation, and the remaining fraction is called ASR, which amounts to ~20–25% of the average input ELV weight. ASR initial specimens were continuously obtained over one week from a crushing and sorting production line for ELVs. Visible bulks of metal to the human eye were sought out from the ASR initial specimens. The characteristics of the ASR specimens were determined by manual sorting and with a Mettler Toledo analytical balance (Changzhou, Jiangsu Province, China), and the results are shown in Table 1.

All specimens were combined and mixed well to ensure a representative sample for the determination, and the uniformity of all specimens regarding the particle size (approximately ≤0.1 mm) was achieved. The reaction mechanism of ASR was unknown, so keeping the initial mass of each sample at a fixed constant for all measurements was safe [35]. Thus, the mass of all samples for the TG experiments was 100 ± 0.1 mg, considering the size of the sample pan and the accuracy of balance in the thermogravimetric analyzer.

### 2.2. Experimental Procedure

**Figure 1:**
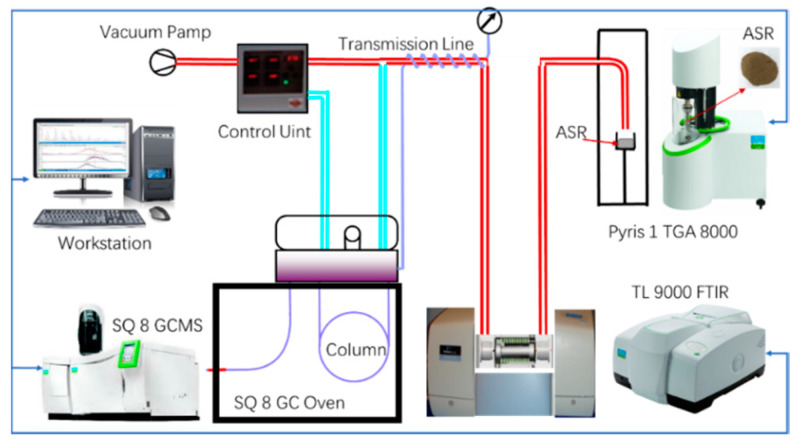
Layout diagram of TG–FTIR–GC–MS (provided by PerkinElmer, MA, USA).

The TG–FTIR–GC/MS coupling technique is widely used in the analysis of organic pyrolysis behaviors. Therefore, we studied the pyrosis behaviors of ASR and its five main components with the TG–FTIR–GC/MS coupling technique. As shown in Figure 1, GC/MS (Perkin-Elmer SQ 8, Waltham, MA, USA) and FTIR (Perkin-Elmer TL 9000, Waltham, MA, USA) equipment was connected with a TG analyzer (Perkin-Elmer TGA 8000, Waltham, MA, USA), and all transmission lines were heated and maintained at 270 °C to prevent the condensation of any volatile disintegration products. Pyris-Manager software was used to analyze the TG and derivate TG (DTG) data, TimeBase Version 3.1.0 (Perkin-Elmer, Waltham, MA, USA) and Spectrum Version 10.5.3 (Perkin-Elmer, Waltham, MA, USA) were applied for the analysis of FTIR spectra data, and the mass spectra data were analyzed using TurboMass Ver 6.0.0 (Perkin-Elmer, Waltham, MA, USA).

The reaction evolution of plastic, textiles, rubber, leather, foam, ASR, and MixASR was recorded for a full range of temperatures from 30 to 800 °C at a constant heating rate of 10 °C/min under atmospheric control with high-purity (99.99%) nitrogen at a rate of 20.0 mL/min. The pressures of pneumatic gas and balance gas were set to 0.1 and 0.2 MPa, respectively. This nitrogen flow rate ensures an inert atmosphere for the sample during the run, whereas a small sample and slow heating rate ensures that the heat transfer limitations can be ignored. Gaseous products from the TG analyzer were directly collected in the gas cell and determined instantaneously using the Fourier transform infrared spectrometer. The resolution in FTIR was 4 cm^−1^, and the scan range was from 4000 to 450 cm^−1^. The FTIR spectra of the gaseous products were obtained continuously with the baseline modified. After the FTIR measurements, the gas products were instantaneously swept to the mass spectrometer. The oven temperature was heated and maintained at 270 °C, and the ionization energy under the electron-impact (EI) of gas volatiles was analyzed at 70 eV. The mass-to-ion ratio (*m*/*z*) ranged from 45 to 400 and was scanned 200 times per minute.

## 3. Results and Discussion

### 3.1. FTIR Analysis of Volatile Products

**Figure 2:**
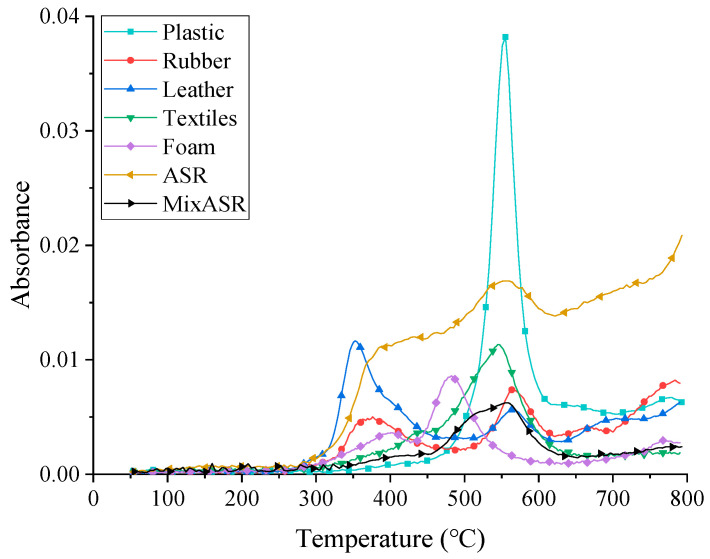
Gram–Schmidt profiles of automobile shredder residue (ASR), MixASR, and its main components.

FTIR is an effective analytical instrument for detecting functional groups and characterizing covalent bonding information. In this study, the Fourier transform infrared spectra of pyrolytic product of plastic, textile, rubber, leather, foam, ASR, and MixASR were recorded to certify the basic chemical group of thermal decomposition product. As shown in Figure 2, Gram–Schmidt profiles of plastic, textile, MixASR, and ASR had only one significant peak, while the Gram–Schmidt profiles of rubber, leather, and foam had two peaks. Below 300 °C, the pyrolysis reactions of ASR, MixASR, or its main components were slow or nonexistent. Over 300 °C, the pyrolysis reactions of rubber, leather, and foam were rapid; the first peak appeared at 346, 322, and 371 °C, respectively, while the second peak appeared at 536, 533, and 452 °C, respectively. Pyrolysis reactions of plastic and textile mainly occurred in the temperature range of 400 to 600 °C, and the only peak appeared at 525 and 515 °C, respectively. Reaching 620 °C, the pyrolysis reaction of plastic, rubber, leather, textile and foam was basically completed, and the curve dropped sharply. The pyrolysis reaction of MixASR mainly occurred in the range of 400–600 °C, and the peak temperature was 526 °C. In addition to the five major organic components, ASR contains 24.6% of other substances in this study, so Gram–Schmidt profiles of ASR and MixASR were very different, but peaks both appeared at 526 °C. The reason is that plastics and fibers accounted for a high proportion of ASR and MixASR, which were the main substances affecting pyrolytic products.

**Figure 3:**
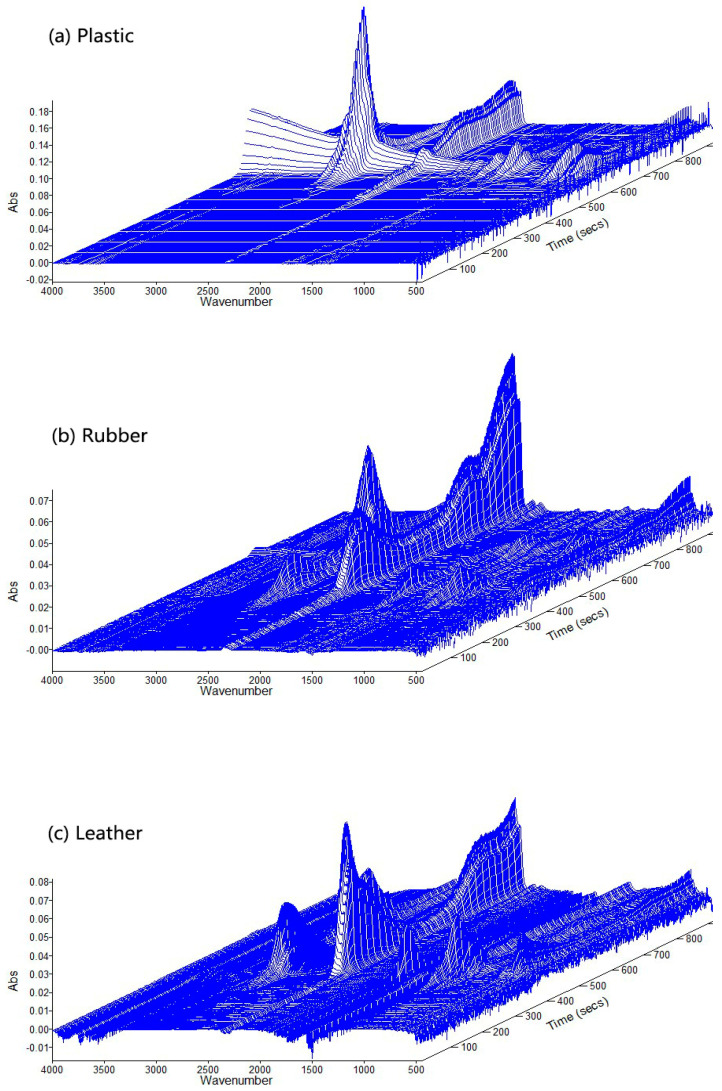

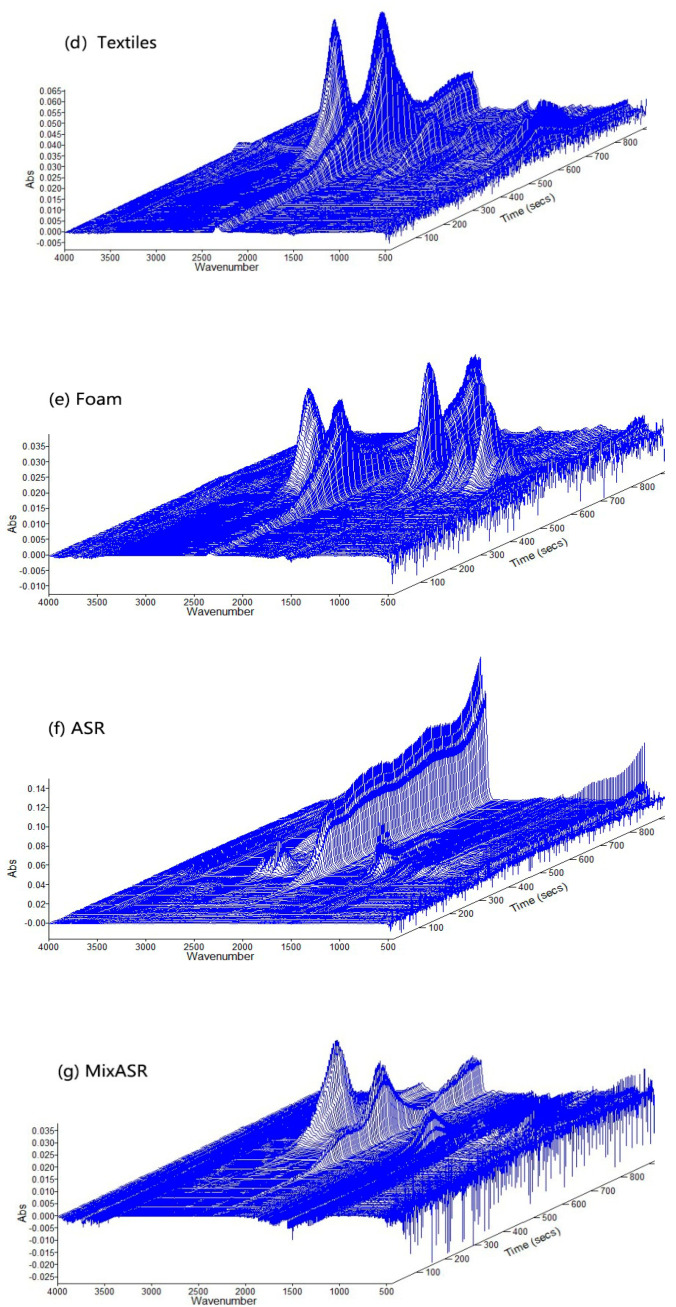
The 3D stack plots of MixASR, ASR, and its main components.

According to the 3D stack plots of MixASR and ASR in Figure 3, the wavenumber ranges of absorption peaks are different. The main absorption peaks in the 3D stack plots of plastic and textiles also appear in the same wavenumber ranges in the 3D stack plots of ASR and MixASR. However, the intensity of the absorption peak at a certain position of MixASR or ASR is not the linear superposition result of the intensities of the absorption peaks at the same position of the five components. In short, the pyrolysis product of ASR is not a simple superposition of the pyrolysis products of its components, but the pyrolysis characteristics of the main components have the greatest influence on the product distribution, especially plastic and textiles.

**Figure 4:**
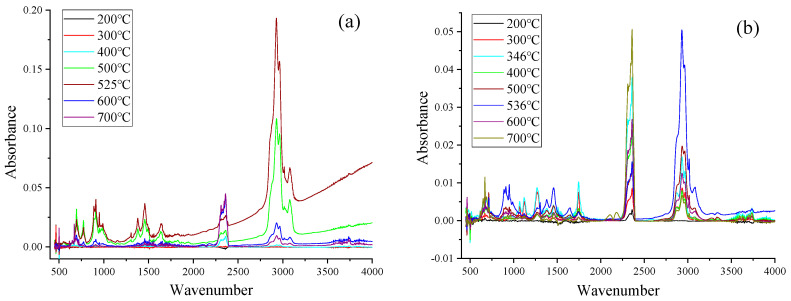

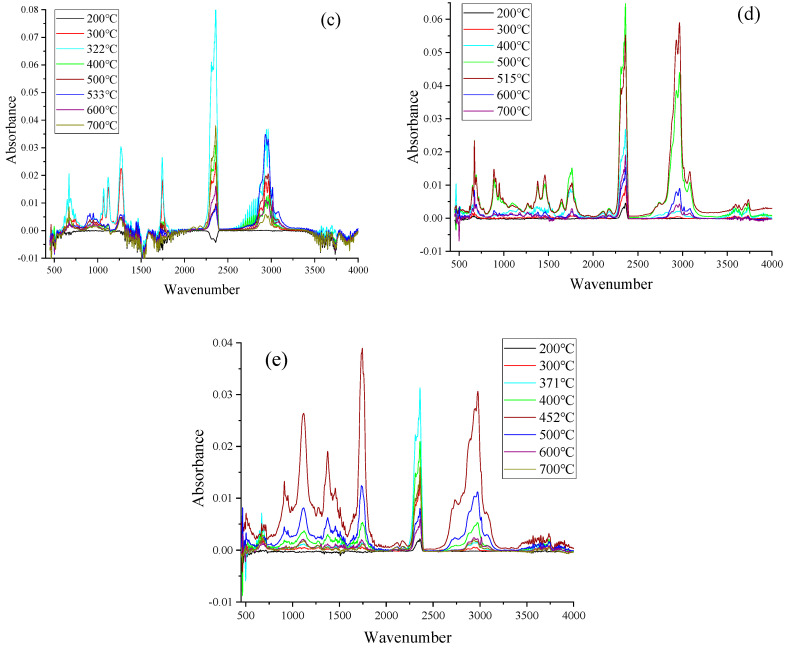
The FTIR spectrum of components of ASR at different pyrolysis temperatures: (**a**) plastic; (**b**) rubber; (**c**) leather; (**d**) textile; and (**e**) foam.

**Figure 5:**
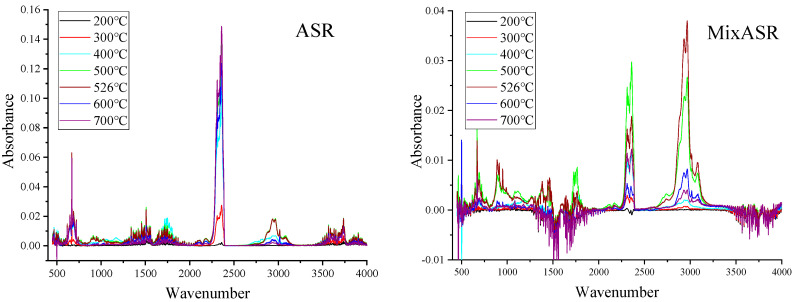
The FTIR spectrum of ASR and MixASR at different pyrolysis temperatures.

With TimeBase and Spectrum software (Perkin-Elmer, Waltham, MA, USA), the Gram–Schmidt profiles of samples were analyzed, and the FTIR spectra of ASR, MixASR, its main components at 200, 300, 400, 500, 600, 700 °C, and temperatures of peaks of Gram–Schmidt profiles are shown in Figure 4 and Figure 5. These functional groups are useful for distinguishing some specific chemical compounds, like CO_2_, alkanes, olefins, benzene, alcohols, and other organic components. According to Figure 4a–e and Figure 5, it can be observed that the locations of the absorption bands were almost the same for the same sample, but the absorbance intensities were different After pyrolysis, the temperature was higher than 300 °C. This indicated that the major categories of volatile products were not affected by the pyrolysis temperature, but the yield of each main product was still affected by the pyrolysis temperature. Strong absorption peaks appeared at 3100–2700, 1700–1550, and 1500–1250 cm^−1^ for ASR and its main components, which were assigned to the stretching of =CH_2_, –CH_3_, –CH_2_, –CH–, and C=C bonds. Furthermore, the absorption intensity of the corresponding macromolecules such as alkanes, aldehydes, and alcohols increased with pyrolysis temperatures and increased sharply at the peak temperatures of the Gram–Schmidt profiles, then sharply decreased. The bands of stretching of =CH–, stretching of –C=C–, and plane –CH– bending –C=CH_2_ were assigned to the alkenes. The strong absorption peaks of 2930, 2850, and 1460 cm^−1^ certify the existence of the methylene (–CH_2_–) group. The formation mechanisms of–CH– and C=C bonds were cleavage of alkyl side chains and β bond scission of alkenes. CO_2_ is represented by the peak between 2395 and 2250 cm^−1^ for ASR and its main components, and the production of CO_2_ increased with temperature, peaked at the first pyrolysis peak temperature, and then decreased sharply. After exceeding 500 °C, peaks appeared between 720–570 and 1050–1150 cm^−1^, which were assigned to the symmetric stretching of C–O. Meanwhile, the CO at 2109–2173 cm^−1^ can also be detected in the entire pyrolysis process. In addition to these substances, each component of ASR had relatively independent functional groups, but which eventually appeared in the mixture FTIR spectrum.

For plastic, the major pyrolytic products were alkanes and olefins, such as styrene, 3-ethyl-3-methyl heptane, and 2,4-dimethyl-1heptene, since the bonds of stretching =CH2, –CH_3_, –CH_2_, –CH– and bending RHC=CH_2_ were assigned. The major pyrolytic products of textiles were CO_2_, alkanes, olefins, benzene, and alcohols, e.g., 2,6-dimethylnonane, styrene, and 2-methyl-1-pentene. Except for CO_2_, alkanes, olefins, and benzene series, the major pyrolytic products of rubber and leather also included alcohols, since the bands of stretching of alcoholic C–OH were assigned to the alcohols, e.g., 1-phenyl-2-propanol. The major pyrolytic products of foam were different; ketones were identified by the bonds of stretching keto C=O, e.g., 1-isopropoxy-propan-2-one. Compared with other components, there are more alkanes and alcohols in foam pyrolysis products.

The major pyrolytic products of ASR and MixASR were much the same, which included alkanes, olefins, and alcohols, and both had dense and indistinguishable weak peaks in the wavenumber range of 1900–1400 cm^−1^. Obviously, many of these products have unstable or weaker chemical bonds, which can be further pyrolyzed under further high-temperature (>800 °C) or catalytic pyrolysis.

### 3.2. GC/MS Analysis of Volatile Products of ASR Pyrolysis

**Figure 6:**
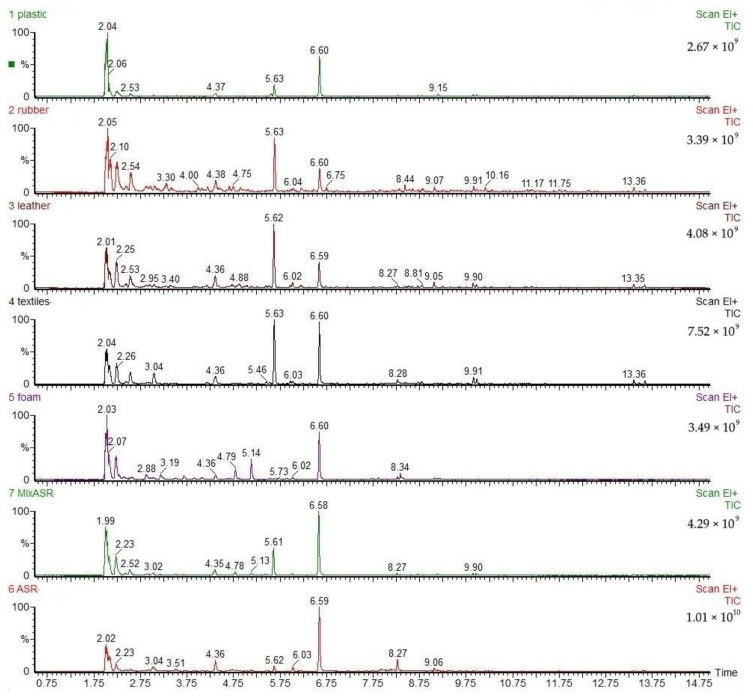
The total ions chromatogram (TIC) of samples at representative temperatures.

In the accurate identification of complicated volatile species, MS analysis is a useful complementary technique for TG–FTIR because TG–FTIR cannot measure homodiatomic species. In order to further accurately analyze the compound types and yields of volatile products of ASR pyrolysis, GC–MS analysis technology was used. The detectable compound was that with a concentration above the detection limit of GC–MS, while the identifiable compound by the NIST library was that with a comparatively large peak area. Since there is no distinguishable peak after 15 min, the chromatogram for the range from 0 to 15 min is shown in Figure 6. Moreover, during the identification of chemical compounds of volatile products, the radicals and functional groups evolved at peaks were based on the results of FTIR analysis. The identification results including compound names and yields of volatile pyrolytic products of ASR and its main components were summarized and are provided in Table 2 and Table 3, and Appendix A. According to the GC–MS analysis results, several points were concluded as follows:

(1) The identified gaseous pyrolytic products were both composed of alkanes, olefins, alcohols, and benzene series, which were consistent with the analysis results of FTIR. The gaseous pyrolytic products of MixASR and ASR were not the simple superposition of pyrolytic gaseous products of its components, but main compounds of MixASR and ASR also appeared in the pyrolytic products of its main components, especially plastic and textile, e.g., styrene, 1-hexene, toluene, ethylbenzene, 2,4-dimethyl-1heptene, and 11-methyl-1-dodecanol.
(2) In the volatile products of the plastics, rubber, leather, textiles, and foam pyrolysis, the total proportions of detectable macromolecular substances were 26.2634, 34.9797, 39.0640, 54.1273, and 39.2296%, respectively. The pyrolysis product of plastic contains 6.0263% olefins, 2.5443% alcohols, 15.25114% benzene series, and very few alkanes. The pyrolysis product of the rubber component contains 13.3232% alkanes, 4.7653% olefins, and 4.6502% alcohols. The leather component pyrolysis product contains 3.3224% alkanes, 7.1102% alkenes, 6.4884% alcohols, and 7.4626% ethers. The pyrolysis products of textiles components contained 1.1139% alkanes, 20.4953% olefins, 7.5055% alcohols, and 15.9611% benzene series. The pyrolysis products of foam components contained 6.8049% alkanes, 12.4046% olefins, 2.4621% ethers, 1.8138% benzene series, and 13.4151% ketones.
(3) The total proportions of detectable macromolecular substances in the volatile products of the original ASR and MixASR pyrolysis were 41.884% and 40.2709%, respectively. The specific detected substances are shown in Table 2 and Table 3. The ASR pyrolysis product contains 3.7385% alkanes, 26.5539% alkenes, and 9.3305% benzene series. The MixASR pyrolysis products contains 0.4045% alkanes, 2.1909% olefins, and 17.739% benzene series. From the comparison of the yield of various pyrolysis products, it can also be seen that the pyrolysis products of ASR or MixASR are not the linear superposition of the pyrolysis products of its main components. This shows once again that the main components of ASR have obvious interactions in the pyrolysis process, and this effect affects the product distribution.
(4) Based on the GC–MS analysis results of ASR and its main components, it can be seen that the yields of olefin and benzene series are high in the pyrolytic products, especially styrene. In pyrolytic products of plastic, textiles, foam, ASR, and MixASR, styrene accounted for 13.62, 11.64, 11.93, 17.18, and 20.68%, respectively. However, these substances are chemically unstable and can be further reacted by improving the process to generate more CO and H_2_.

### 3.3. Analysis, Summary, and Discussion

According to the Py–FTIR–GC–MS analysis, the pyrolysis process of ASR could be concluded as follows. Firstly, small molecule alkenes and cycloalkenes were generated from the degradation of ASR due to chain scission at low temperature. Then, the released alkenes and cycloalkenes underwent a series of reactions to form benzene and benzene derivatives as the pyrolysis temperature increased. The main pyrolysis products were concentrated in alkenes, olefins, alcohols, and aromatic hydrocarbons. The yields of volatile products of ASR pyrolysis varied greatly with changing pyrolysis temperature. Considering that plastic and textiles account for a high proportion of ASR, and other organic matter is affected by them during the pyrolysis process [21], the design of pyrolysis temperature can refer to the pyrolysis characteristics of plastics and fibers. Since most volatile products are chemically unstable, more syngas with a greater calorific value could be obtained by catalytic pyrolysis and gasification [36,37,38].

## 4. Conclusions

In this work, efforts were made to investigate the pyrolysis characteristics of ASR and its main components (i.e., plastic, textile, leather, rubber, and foam) through TG–FTIR-GC–MS. The mass variation of samples and the gaseous products were measured simultaneously. Comparing the results of FTIR and GC–MS analysis, good consistency between these two approaches was observed, and the main conclusions can be summarized as follows:

(1) The main volatile products of ASR and its main components are alkanes, olefins, alcohols, and benzene series, and their proportions in the pyrolysis products are 3.7385, 26.5539, and 9.3305%, respectively. Many of these volatile products have unstable or weaker chemical bonds, such as =CH–, =CH_2_, –C=C–, and –C=CH_2_. Hence, more syngas can be obtained with further high-temperature (>800 °C) pyrolysis [39,40]. Catalytic pyrolysis and gasification are important research directions for obtaining syngas with a greater calorific value [36,37,38].
(2) According to the Gram–Schmidt profiles and the 3D stack plots of MixASR, ASR, and its main components, the pyrolysis product of ASR is not a simple superposition of the pyrolysis products of its components, but the pyrolysis characteristics of the main components have the greatest influence on the product distribution, especially plastic and textiles. Some hazardous gas exists in pyrolytic products of ASR, such as benzene and toluene, which are harmful to the human body and environment. Therefore, the elimination of toxic and hazardous substances must be considered in the design of pyrolysis process.

In brief, the TG–FTIR–GC–MS coupling technique provided a deeper insight into the understanding of pyrolysis behaviors of ASR. This is helpful for the design of further pyrolysis processes for obtaining more syngas with higher heating value. It could also provide basic information for an industrial pilot or industrialization of the ASR or polymer pyrolysis process.

## Figures and Tables

**Table 1:** Material compositions of the experimental specimens.

Sample	Material Composition (wt.%)						
	Metals	Plastics	Textiles	Leather	Rubber	Foam	Others
ASR	3.0	39.7	28.1	3.3	2.2	2.1	21.6
MixASR	0	53	37	3	4	3	0

**Table 2:** GC–MS analysis results of the original ASR pyrolysis volatiles.

No.	Peaks	Formula	Compound	CAS Number	Yield/%
1	2.226	C_8_H_16_O	1-prop-2-enoxypentane	23186-70-1	2.8681
2	2.445	C_3_H_9_NO	1-aminopropan-2-ol	78-96-6	0.4912
3	2.544	C_6_H_12_	1-hexene	592-41-6	0.8056
		C_6_H_12_	4-methyl-1-Pentene	691-37-2	
		C_6_H_13_Cl	2-chlorohexane	638-28-8	
4	2.771	C_4_H_8_O	tetrahydrofuran	109-99-9	0.2440
		C_5_H_10_O_2_	4-ethyl-1,3-dioxolane	29921-38-8	
5	3.037	C_6_H_11_ClO	α-chlorohexanal	762-39-0	2.6292
6	3.508	C_7_H_12_O_3_	2-methylacrylic acid 3-hydroxypropyl ester	2761-09-3	0.8626
		C_7_H_12_O_3_	2-hydroxypropyl methacrylate	923-26-2	
7	4.364	C_7_H_8_	toluene	108-88-3	4.1164
8	4.798	C_8_H_18_O	2,3-dimethylhexan-3-ol	4166-46-5	0.2205
		C_6_H_14_O	4-Methyl-2-pentanol	108-11-2	
9	5.147	C_6_H_12_O_2_	1-isopropoxy-propan-2-one	42781-12-4	0.3793
10	5.623	C_9_H_18_	2,4-dimethyl-1heptene	19549-87-2	1.6540
11	6.026	C_8_H_10_	ethylbenzene	100-41-4	1.2742
			o-Xylene	95-47-6	
12	6.593	C_8_H_8_	annulene	629-20-9	20.6849
			benzocyclobutene	694-87-1	
			styrene	100-42-5	
13	7.205	C_9_H_12_	cumene	98-82-8	0.0749
14	7.614	C_9_H_10_	prop-2-enylbenzene	300-57-2	0.1990
15	8.127	C_6_H_6_O	phenol	108-95-2	0.3113
16	8.181	C_8_H_14_O_2_	butyl methacrylate	97-88-1	0.2616
17	8.274	C_9_H_10_	2-phenyl-1-propene	98-83-9	3.0575
18	8.717	C_14_H_30_	7-methyltridecane	26730-14-3	0.0597
		C_10_H_22_	3-ethyl-3-methyl heptane	17302-01-1	
19	9.06	C_8_H_18_O	2-ethylhexan-1-ol	104-76-7	0.8311
			2-propylpentan-1-ol	58175-57-8	
20	9.142	C_8_H_16_	3,4,4-trimethyl-2-pentene	598-96-9	0.2199
			2,4,4-trimethyl-2-pentene	107-40-4	
21	9.36	C_10_H_12_	4-phenyl-1-butene	768-56-9	0.1319
22	9.601	C_10_H_12_	alpha,p-dimethylstyrene	1195-32-0	0.2256
23	9.754	C_8_H_8_O	acetophenone	98-86-2	0.0718
24	9.907	C_13_H_28_O	11-methyl-1-dodecanol	85763-57-1	0.2098

**Table 3:** GC–MS analysis results of the MixASR pyrolysis volatiles.

No.	Peaks	Formula	Compound	CAS Number	Yield/%
1	2.229	C_6_H_12_O	1-prop-2-enoxypropane	1471-03-0	8.1671
2	2.431	C_7_H_10_O_6_	methanetricarboxylic acid, 1,1,1-trimethyl ester	1186-73-8	0.4723
3	2.521	C_6_H_12_	2-methyl-1-pentene	763-29-1	1.9019
			1-hexene	592-41-6	
4	4.347	C_9_H_12_O	1-phenyl-2-propanol	698-87-3	2.0945
5	4.784	C_6_H_14_O	4-methyl-2-pentanol	108-11-2	0.8483
6	5.13	C_6_H_12_O_2_	1-isopropoxy-propan-2-one	42781-12-4	0.7124
7	5.609	C_9_H_18_	2,4-dimethyl-1heptene	19549-87-2	6.9013
8	6.0009	C_8_H_10_	ethylbenzene	100-41-4	0.2366
			o-xylene	95-47-6	
9	6.579	C_8_H_8_	styrene	100-42-5	17.1758
10	8.266	C_9_H_10_	2-phenyl-1-propene	98-83-9	0.3269
11	8.702	C_10_H_22_	3-ethyl-3-methyl heptane	17302-01-1	0.4045
		C_11_H_24_	2,6-dimethylnonane	17302-28-2	
		C_14_H_30_	dodecane,4,6-dimethyl	61141-72-8	
12	9.136	C_10_H_16_	(3R)-(+)-isosylvestren	1461-27-4	0.2890
13	9.902	C_13_H_28_O	11-methyl-1-dodecanol	85763-57-1	0.7403

## Abbreviations

The following abbreviations are used in this manuscript:

ASR	Automobile shredder residue
MixASR	Mixtures of plastic, textiles, leather, rubber, and foam
Py	Pyrolysis
FTIR	Fourier transform infrared spectrometry
GC/MS	Gas chromatography–mass spectrometry
PVC	Polyvinyl chloride
TGA	Thermogravimetry analysis
HHVs	Higher heating values

## Appendix A

**Table A1:** GC–MS analysis results of the plastic pyrolysis volatiles.

No.	Peaks	Formula	Compound	CAS Number	Yield/%
1	2.249	C_6_H_10_O	6-methyl-3,6-dihydro-2H-pyran	55230-25-6	3.5802
2	2.533	C_6_H_12_	2-methyl-1-pentene	763-29-1	1.0352
3	3.517	C_5_H_8_O_2_	methyl methacrylate	80-62-6	0.1645
3	4.37	C_9_H_12_O	1-phenyl-2-propanol	698-87-3	1.5010
5	5.558	C_8_H_12_	4-vinyl-1-cyclohexene	100-40-3	0.5372
6	5.629	C_9_H_18_	2,4-dimethyl-1heptene	19549-87-2	3.6554
		C_8_H_16_	5-methylhept-1-ene	13151-04-7	
		C_10_H_20_	3,7-dimethyloct-1-ene	4984/1/4	
7	6.601	C_8_H_8_	annulene	629-20-9	13.6182
			benzocyclobutene	694-87-1	
			styrene	100-42-5	
8	8.277	C_9_H_10_	2-phenyl-1-propene	98-83-9	0.1321
9	8.717	C_10_H_22_	3-ethyl-3-methyl heptane	17302-01-1	0.1538
10	8.793	C_11_H_24_	2,6-dimethylnonane	17302-28-2	0.1761
11	9.148	C_10_H_16_	(3R)-(+)-Isosylvestren	1461-27-4	0.6663
12	9.91	C_13_H_28_O	11-methyl-1-dodecanol	85763-57-1	0.3317
13	9.978	C_13_H_28_O	11-methyl-1-dodecanol	85763-57-1	0.2899
14	13.352	C_13_H_28_O	11-methyl-1-dodecanol	85763-57-1	0.1570
15	13.474	C_13_H_28_O	11-methyl-1-dodecanol	85763-57-1	0.0933
16	13.596	C_13_H_28_O	11-methyl-1-dodecanol	85763-57-1	0.1712

**Table A2:** GC–MS analysis results of the rubber pyrolysis volatiles.

No.	Peaks	Formula	Compound	CAS Number	Yield/%
1	2.249	C_5_H_12_	2-methylbutane	78-78-4	7.2044
2	2.439	C_6_H_14_O	2-methyl-1-pentanol	105-30-6	0.5324
3	2.544	C_6_H_12_	1-hexene	592-41-6	3.9098
		C_6_H_12_	methylcyclopentane	96-37-7	
		C_6_H_13_Cl	2-chlorohexane	638-28-8	
4	2.958	C_6_H_10_	bipropenyl	592-46-1	0.5624
5	3.054	C_6_H_6_	benzene	71-43-2	0.6028
6	3.304	C_7_H_14_	1-heptene	592-76-7	1.8165
7	3.406	C_7_H_16_	heptane	142-82-5	0.4222
8	3.599	C_7_H_12_	2,4-dimethyl-1,3-pentadiene	1000-86-8	0.1327
9	3.999	C_7_H_10_	1-methyl-1,4-cyclohexadiene	4313-57-9	0.5747
10	4.197	C_10_H_16_O	3-hydroxy-2-methyl-6-methylene-1,7-octadiene	22459-10-5	0.6420
11	4.376	C_9_H_12_O	1-phenyl-2-propanol	698-87-3	1.8206
12	4.662	C_8_H_16_	2-methyl-1-heptene	15870-10-7	0.4028
13	4.747	C_8_H_16_	oct-1-ene	111-66-0	0.6065
		C_8_H_18_O	octan-1-ol	111-87-5	
14	4.9	C_8_H_18_	octane	111-65-9	0.4613
15	5.065	C_8_H_14_O	oct-2-yn-1-ol	20739-58-6	0.1743
16	5.635	C_9_H_18_	2,4-dimethyl-1-heptene	19549-87-2	6.3153
17	6.037	C_8_H_10_	ethylbenzene	100-41-4	0.2325
18	6.21	C_12_H_16_O	2-phenyl-hex-5-en-3-ol	77383-06-3	0.5773
19	6.601	C_12_H_14_O_2_	2,2-dimethyl-5-phenyloxolan-3-one	63678-00-2	3.2998
20	6.752	C_9_H_20_	nonane	111-84-2	0.3576
21	7.903	C_21_H_26_O_2_	benzoic acid-(2,4-di-tert-butyl-phenyl ester)	39000-49-2	0.1512
22	8.439	C_10_H_20_	1-decene	872-05-9	0.5524
23	8.595	C_10_H_22_	decane	124-18-5	0.2374
24	8.722	C_10_H_22_	3,3,5-trimethylheptane	7154-80-5	0.1590
25	8.819	C_16_H_34_	hexadecane	544-76-3	0.4068
26	9.068	C_8_H_18_O	2-ethylhexan-1-ol	104-76-7	0.4132
27	9.261	C_10_H_12_O	2-phenylbut-3-en-1-ol	6052-63-7	0.1071
28	9.91	C_13_H_28_O	11-methyl-1-dodecanol	85763-57-1	0.4746
29	10.163	C_11_H_22_	1-undecene	821-95-4	0.3116
30	10.302	C_11_H_24_	undecane	1120-21-4	0.1645
31	10.69	C_15_H_32_O	3,7,11-trimethyldodecan-1-ol	6750-34-1	0.1266
32	10.798	C_20_H_40_O	phytol	150-86-7	0.0440
33	11.064	C_16_H_26_O_3_	2-dodecen-1-ylsuccinic anhydride	19780-11-1	0.0765
		C_10_H_20_O	cyclohexanemethanol, 2,4,6-trimethyl-	13702-56-2	
34	11.172	C_13_H_18_O	ether,isopropyl 2-benzyl-2-propenyl	900152-79-5	0.1764
35	11.645	C_12_H_24_	5-undecene,2-methyl-,(Z)-	74630-63-0	0.0857
36	11.753	C_12_H_24_	1-dodecene	112-41-4	0.1798
37	11.878	C_17_H_37_N	1-aminoheptadecane	4200-95-7	0.0804
38	13.355	C_18_H_38_O	2-hexyldodecan-1-ol	110225-00-8	0.5847
		C_13_H_28_O	11-methyl-1-dodecanol	85763-57-1	

**Table A3:** GC–MS analysis results of the leather pyrolysis volatiles.

No.	Peaks	Formula	Compound	CAS Number	Yield/%
1	2.246	C_8_H_16_O	1-prop-2-enoxypentane	23186-70-1	7.4626
2	2.439	C_5_H_10_O	3-penten-2-ol	1569-50-2	0.7402
3	2.53	C_6_H_12_	2-methyl-1-pentene	763-29-1	2.7042
			1-hexene	592-41-6	
4	2.952	C_6_H_10_	bipropenyl	592-46-1	0.4749
5	3.052	C_6_H_6_	benzene	71-43-2	0.5936
6	3.307	C_7_H_16_O	(s)-3,4-dimethylpentanol	900143-83-9	0.3582
7	3.4	C_7_H_16_	heptane	142-82-5	0.5957
8	4.361	C_9_H_12_O	1-phenyl-2-propanol	698-87-3	2.6530
9	4.722/4.88/5.039	C_8_H_16_	trans-1-butyl-2-methylcyclopropane	38851-70-6	2.5389
			cis-1-butyl-2-methylcyclopropane	38851-69-3	
			trans-2-octene	13389-42-9	
			trans-4-octene	14850-23-8	
		C_8_H_17_Cl	4-chlorooctane	999-07-5	
			3-chlorooctane	1117-79-9	
10	5.618	C_9_H_18_	2,4-dimethyl-1-heptene	19549-87-2	9.8874
11	6.017	C_8_H_10_	ethylbenzene	100-41-4	1.1988
12	6.59	C_8_H_8_	styrene	100-42-5	4.8862
13	7.894	C_9_H_12_	3-ethyltoluene	620-14-4	0.2492
14	8.215	C_9_H_12_	1-ethyl-4-methylbenzene	622-96-8	0.1529
			1-ethyl-2-methylbenzene	611-14-3	
			3-ethyltoluene	620-14-4	
15	8.269	C_9_H_10_	2-phenyl-1-propene	98-83-9	0.2948
16	8.427	C_14_H_28_	(e)-tetradec-3-ene	41446-68-8	0.1934
17	8.586	C_10_H_22_	decane	124-18-5	0.1247
18	8.708	C_10_H_22_	3-ethyl-3-methylheptane	17302-01-1	0.1877
19	8.807	C_20_H_17_NO_2_	6-methyl-dodecane	6044-71-9	0.4697
20	9.054	C_8_H_18_O	2-ethylhexan-1-ol	104-76-7	0.8314
21	9.244	C_9_H_10_	indane	496-11-7	0.2027
22	9.902/9.973/13.349	C_13_H_28_O	11-methyl-1-dodecanol	85763-57-1	2.2638

**Table A4:** GC–MS analysis results of the textiles pyrolysis volatiles.

No.	Peaks	Formula	Compound	CAS Number	Yield/%
1	2.238	C_4_H_11_NO	O-(2-methylpropyl)hydroxylamine	5618-62-2	6.9797
2	2.45	C_6_H_14_O	2,3-dimethylbutyl alcohol	19550-30-2	0.4687
3	2.533	C_6_H_12_	2-methyl-1-pentene	763-29-1	3.7191
			1-hexene	592-41-6	
4	3.043	C_6_H_6_	benzene	71-43-2	3.4949
5	4.191	C_8_H_16_	2,3-dimethylhex-1-ene	16746-86-4	0.3236
			2,5-dimethyl-2-hexene	3404-78-2	
			(E)-2,3-dimethylhex-3-ene	7145-23-5	
6	4.364	C_9_H_12_O	1-phenyl-2-propanol	698-87-3	2.9873
7	5.133	C_9_H_18_	cis-1,1,3,4-tetramethylcyclopentane	53907-60-1	0.2179
8	5.272	C_9_H_20_	2,4-dimethyl-heptane	2213-23-2	0.0875
9	5.459	C_10_H_18_	isocitronellene	85006-04-8	0.3957
10	5.629	C_8_H_16_	5-methylhept-1-ene	13151-04-7	16.0668
11	5.972	C_9_H_18_	1,3,5-trimethyl-cyclohexane	1839-63-0	0.3468
12	6.026	C_8_H_10_	ethylbenzene	100-41-4	0.3153
13	6.599	C_8_H_8_	styrene	100-42-5	11.6357
14	6.973	C_10_H_18_	2,5-dimethyl-1,trans-6-octadien	68702-25-0	0.1709
15	7.208	C_9_H_12_	cumene	98-82-8	0.0322
16	7.616	C_9_H_10_	prop-2-enylbenzene	300-57-2	0.0838
17	7.767	C_13_H_18_O_2_	phenylacetic acid isoamyl ester	102-19-2	0.0935
18	7.92	C_7_H_6_O	benzaldehyde	100-52-7	0.1585
19	8.277	C_9_H_10_	2-phenyl-1-propene	98-83-9	0.9701
20	8.43	C_16_H_32_	hexadec-3-ene	34303-81-6	0.3858
21	8.717/8.787	C_14_H_30_	dodecane,4,6-dimethyl	61141-72-8	0.6796
		C_11_H_24_	2,6-dimethylnonane	17302-28-2	
22	9.907/9.981	C_13_H_28_O	11-methyl-1-dodecanol	85763-57-1	2.3873
23	10.236	C_8_H_8_O_2_	methyl benzoate	93-58-3	0.0960
24	10.684/10.792	C_15_H_32_O	3,7,11-trimethyldodecan-1-ol	6750-34-1	0.1598
25	10.894	C_9_H_8_O_2_	vinyl benzoate	769-78-8	0.1427
26	11.064	C_10_H_20_O	dihydoisocyclogeraniol	13702-56-2	0.1177
27	11.45	C_9_H_10_O_2_	ethyl benzoate	93-89-0	0.1083
28	13.355/13.474/13.590	C_13_H_28_O	11-methyl-1-dodecanol	85763-57-1	1.5021

**Table A5:** GC–MS analysis results of the foam pyrolysis volatiles.

No.	Peaks	Formula	Compound	CAS Number	Yield/%
1	2.226	C_6_H_12_O_2_	1-isopropoxy-propan-2-one	42781-12-4	6.6008
2	2.399	C_3_H_6_N_2_O_2_	cycloserine	68-41-7	0.6897
3	2.884	C_6_H_14_O	1-propan-2-yloxypropane	627-08-7	1.3509
4	3.188	C_6_H_12_O	2,2,3,3-tetramethyloxirane	5076-20-0	1.9050
5	3.502	C_17_H_34_O_2_	2-(tetradecoxymethyl)oxirane	38954-75-5	0.4383
6	3.689	C_6_H_12_O_2_	2,2,4-Trimethyl-1,3-dioxolane	1193-11-9	1.2745
7	3.908	C_6_H_10_O_2_	4-methylpentane-2,3-dione	7493-58-5	0.3843
8	4.067	C_11_H_22_O_2_	2-ethylhexyl glycidyl ether	2461-15-6	0.7434
9	4.361	C_7_H_8_	toluene	108-88-3	1.3903
10	4.79	C_8_H_18_O_2_	1-(1-propoxyethoxy)propane	105-82-8	2.6692
11	5.135	C_6_H_12_O_2_	1-isopropoxy-propan-2-one	42781-12-4	5.3007
12	5.532	C_6_H_12_O_2_	2,2,4-trimethyl-1,3-dioxolane	1193-11-9	0.1558
13	5.62	C_10_H_20_O	decanal	112-31-2	0.2558
14	5.728	C_6_H_13_NO	hexanamide	628-02-4	0.3094
15	6.023	C_8_H_10_	ethylbenzene	100-41-4	0.4235
16	6.599	C_8_H_8_	annulene	629-20-9	11.9324
			styrene	100-42-5	
17	7.078	C_7_H_16_O	4-methylhexan-3-ol	615-29-2	0.0498
18	7.738	C_7_H_16_O	2-propan-2-yloxybutane	18641-81-1	0.3678
19	7.869	C_8_H_18_O	4-methyl-3-heptanol	14979-39-6	0.2974
20	8.274	C_9_H_10_	2-phenyl-1-propene	98-83-9	0.4722
21	8.337	C_6_H_12_O_2_	5-methoxypentan-2-one	17429-04-8	1.5136
22	9.071	C_8_H_18_O	6-methyl-1-heptanol	1653-40-3	0.3426
23	9.13	C_8_H_17_Cl	3-chlorooctane	1117-79-9	0.3620

## References

[1] Nourreddine M. (2007). Recycling of auto shredder residue. J. Hazard. Mater..

[2] Fiore S., Ruffino B., Zanetti M.C. (2012). Automobile Shredder Residues in Italy: Characterization and valorization opportunities. Waste Manag..

[3] Cossu R., Fiore S., Lai T., Luciano A., Mancini G., Ruffino B., Viotti P., Zanetti M.C. (2014). Review of Italian experience on automotive shredder residue characterization and management. Waste Manag..

[4] Ruffino B., Fiore S., Zanetti M.C. (2014). Strategies for the enhancement of automobile shredder residues (ASRs) recycling: Results and cost assessment. Waste Manag..

[5] Ahmed N., Wenzel H., Hansen J.B. (2014). Characterization of Shredder Residues generated and deposited in Denmark. Waste Manag..

[6] Cossu R., Lai T. (2015). Automotive shredder residue (ASR) management: An overview. Waste Manag..

[7] Morselli L., Santini A., Passarini F., Vassura I. (2010). Automotive shredder residue (ASR) characterization for a valuable management. Waste Manag..

[8] Passarini F., Ciacci L., Santini A., Vassura I., Morselli L. (2012). Auto shredder residue LCA: Implications of ASR composition evolution. J. Clean. Prod..

[9] Mancini G., Viotti P., Luciano A., Fino D. (2014). On the ASR and ASR thermal residues characterization of full scale treatment plant. Waste Manag..

[10] Vermeulen I., Van Caneghem J., Block C., Baeyens J., Vandecasteele C. (2011). Automotive shredder residue (ASR): Reviewing its production from end-of-life vehicles (ELVs) and its recycling, energy or chemicals’ valorisation. J. Hazard. Mater..

[11] Roy C., Chaala A. (2001). Vacuum pyrolysis of automobile shredder residues. Resour. Conserv. Recycl..

[12] Zolezzi M., Nicolella C., Ferrara S., Iacobucci C., Rovatti M. (2004). Conventional and fast pyrolysis of automobile shredder residues (ASR). Waste Manag..

[13] Santini A., Passarini F., Vassura I., Serrano D., Dufour J., Morselli L. (2012). Auto shredder residue recycling: Mechanical separation and pyrolysis. Waste Manag..

[14] Harder M.K., Forton O.T. (2007). A critical review of developments in the pyrolysis of automotive shredder residue. J. Anal. Appl. Pyrolysis.

[15] Ni F.J., Chen M. (2015). Research on ASR in China and its energy recycling with pyrolysis method. J. Mater. Cycles Waste Manag..

[16] Haydary J., Susa D., Gelinger V., Čacho F. (2016). Pyrolysis of automobile shredder residue in a laboratory scale screw type reactor. J. Environ. Chem. Eng..

[17] Mayyas M., Pahlevani F., Handoko W., Sahajwalla V. (2016). Preliminary investigation on the thermal conversion of automotive shredder residue into value-added products: Graphitic carbon and nano-ceramics. Waste Management.

[18] Anzano M., Collina E., Piccinelli E., Lasagni M. (2017). Lab-scale pyrolysis of the Automotive Shredder Residue light fraction and characterization of tar and solid products. Waste Manag..

[19] Notarnicola M., Cornacchia G., De Gisi S., Di Canio F., Freda C., Garzone P., Martino M., Valerio V., Villone A. (2017). Pyrolysis of automotive shredder residue in a bench scale rotary kiln. Waste Manag..

[20] Evangelopoulos P., Sophonrat N., Jilvero H., Yang W. (2018). Investigation on the low-temperature pyrolysis of automotive shredder residue (ASR) for energy recovery and metal recycling. Waste Manag..

[21] Yang B., Chen M. (2020). Influence of Interactions among Polymeric Components of Automobile Shredder Residue on the Pyrolysis Temperature and Characterization of Pyrolytic Products. Polymers.

[22] He M., Hu Z., Xiao B., Li J., Guo X., Luo S., Yang F., Feng Y., Yang G., Liu S. (2009). Hydrogen-rich gas from catalytic steam gasification of municipal solid waste (MSW): Influence of catalyst and temperature on yield and product composition. Int. J. Hydrog. Energy.

[23] He M., Xiao B., Liu S., Hu Z., Guo X., Luo S., Yang F. (2010). Syngas production from pyrolysis of municipal solid waste (MSW) with dolomite as downstream catalysts. J. Anal. Appl. Pyrolysis.

[24] Blanco P.H., Wu C., Onwudili J.A., Williams P.T. (2013). Characterization and evaluation of Ni/SiO2 catalysts for hydrogen production and tar reduction from catalytic steam pyrolysis-reforming of refuse derived fuel. Appl. Catal. B Environ..

[25] He M., Xiao B., Liu S., Guo X., Luo S., Xu Z., Feng Y., Hu Z. (2009). Hydrogen-rich gas from catalytic steam gasification of municipal solid waste (MSW): Influence of steam to MSW ratios and weight hourly space velocity on gas production and composition. Int. J. Hydrog. Energy.

[26] Acomb J.C., Wu C., Williams P.T. (2014). Control of steam input to the pyrolysis-gasification of waste plastics for improved production of hydrogen or carbon nanotubes. Appl. Catal. B Environ..

[27] Donaj P., Blasiak W., Yang W., Forsgren C. (2011). Conversion of microwave pyrolysed ASR’s char using high temperature agents. J. Hazard. Mater..

[28] Lin K.-S., Chowdhury S., Wang Z.-P. (2010). Catalytic gasification of automotive shredder residues with hydrogen generation. J. Power Sources.

[29] Kai X., Li R., Yang T., Shen S., Ji Q., Zhang T. (2017). Study on the co-pyrolysis of rice straw and high density polyethylene blends using TG-FTIR-MS. Energy Convers. Manag..

[30] Wu J., Chen T., Luo X., Han D., Wang Z., Wu J. (2014). TG/FTIR analysis on co-pyrolysis behavior of PE, PVC and PS. Waste Manag..

[31] Zhang J., Chen T., Wu J., Wu J. (2015). TG-MS analysis and kinetic study for thermal decomposition of six representative components of municipal solid waste under steam atmosphere. Waste Manag..

[32] Singh S., Wu C., Williams P.T. (2012). Pyrolysis of waste materials using TGA-MS and TGA-FTIR as complementary characterisation techniques. J. Anal. Appl. Pyrolysis.

[33] Guo Q.J., Zhang X., Li C., Liu X.M., Li J.H. (2012). TG-MS study of the thermo-oxidative behavior of plastic automobile shredder residues. J. Hazard. Mater..

[34] Mayyas M., Pahlevani F., Bucknall M., Maroufi S., You Y., Liu Z., Sahajwalla V. (2017). Thermocatalytic Conversion of Automotive Shredder Waste and Formation of Nanocarbons as a Process Byproduct. Acs Sustain. Chem. Eng..

[35] Gao Z., Nakada M., Amasaki I. (2001). A consideration of errors and accuracy in the isoconversional methods. Thermochim. Acta.

[36] Guan Y., Luo S., Liu S., Xiao B., Cai L. (2009). Steam catalytic gasification of municipal solid waste for producing tar-free fuel gas. Int. J. Hydrog. Energy.

[37] Arregi A., Amutio M., Lopez G., Artetxe M., Alvarez J., Bilbao J., Olazar M. (2017). Hydrogen-rich gas production by continuous pyrolysis and in-line catalytic reforming of pine wood waste and HDPE mixtures. Energy Convers. Manag..

[38] Duman G., Yanik J. (2017). Two-step steam pyrolysis of biomass for hydrogen production. Int. J. Hydrog. Energy.

[39] Al-Salem S.M., Antelava A., Constantinou A., Manos G., Dutta A. (2017). A review on thermal and catalytic pyrolysis of plastic solid waste (PSW). J. Environ. Manag..

[40] Almeida D., Marques M.d.F. (2016). Thermal and Catalytic Pyrolysis of Plastic Waste. Polímeros.

